# Clinical presentation, genotype–phenotype correlations, and outcome of pancreatic neuroendocrine tumors in Von Hippel–Lindau syndrome

**DOI:** 10.1007/s12020-021-02752-8

**Published:** 2021-05-25

**Authors:** F. Penitenti, L. Landoni, M. Scardoni, M. L. Piredda, S. Cingarlini, A. Scarpa, M. D’Onofrio, D. Girelli, M. V. Davi

**Affiliations:** 1 Department of Medicine, Section of Endocrinology, ENETS Center of Excellence, University and Hospital Trust of Verona, Verona, Italy; 2 Department of Surgery, The Pancreas Institute, ENETS Center of Excellence, University and Hospital Trust of Verona, Verona, Italy; 3 Department of Diagnostics and Public Health, Section of Pathology, ENETS Center of Excellence, University and Hospital Trust of Verona, Verona, Italy; 4 ARC-Net Research Center, University and Hospital Trust of Verona, Verona, Italy; 5 Department of Medicine, Section of Oncology, ENETS Center of Excellence, University and Hospital Trust of Verona, Verona, Italy; 6 Department of Radiology, ENETS Center of Excellence, University and Hospital Trust of Verona, Verona, Italy; 7 Department of Medicine, Section of Internal Medicine, ENETS Center of Excellence, University and Hospital Trust of Verona, Verona, Italy

**Keywords:** Von Hippel–Lindau syndrome, Pancreatic neuroendocrine tumors, Genotype–phenotype correlations, Clinical presentation

## Abstract

**Purpose:**

Data regarding the clinical management and follow-up of pancreatic neuroendocrine tumors (PanNETs) associated with Von Hippel–Lindau (VHL) syndrome are limited. This study aimed to assess clinical presentation, genotype–phenotype correlations, treatment and prognosis of PanNETs in a series of VHL syndrome patients.

**Methods:**

Retrospective analysis of data of patients observed between 2005 and 2020.

**Results:**

Seventeen patients, including 12 probands and 5 relatives (mean age 30.8 ± 18.4; 7 males), were recruited. PanNETs were found in 13/17 patients (77.5%) at a median age of 37 years: 4/13 (30.7%) at the time of VHL diagnosis and 9 (69.3%) during follow up. Six (46.1%) PanNET patients underwent surgery, whereas seven were conservatively treated (mean tumor diameter: 40 ± 10.9 vs. 15 ± 5.3 mm respectively). Four patients (30.7%) had lymph node metastases and a mean tumor diameter significantly larger than the nonmetastatic PanNETs (44.2 ± 9.3 vs. 17.4 ± 7 mm, *p* = 0.00049, respectively). Five (83.3%) operated patients had stable disease after a median follow up of 3 years whereas one patient showed liver metastases. Six (85.7%) non-resected PanNETs were stable after a median follow-up of 2 years, whereas one patient developed a new small PanNET and a slight increase in diameter of a pre-existing PanNET. No correlation was found between the type of germline mutation and malignant behavior of PanNETs.

**Conclusions:**

PanNETs are a common disease of the VHL syndrome and can be the presenting feature. Tumor size rather than genetic mutation is a prognostic factor of malignancy.

## Introduction

Von Hippel–Lindau (VHL) syndrome is a rare autosomal dominant disease due to a germline mutation of the *VHL* gene, located at chromosome 3p25-26. The incidence is 1:36,000 livebirths and the mean age of presentation is around 26 years [1]. The genetic alteration is the loss of the tumor suppressor function of the *VHL* gene, which encodes a protein participating in the oxygen sensing system [1]. VHL syndrome is characterized by benign and malignant neoplasms, including retinal/central nervous system (CNS) haemangioblastomas (HBA) (60–84%), pheocromocytomas (10–20%), renal cysts and carcinomas (RCC) (25–60%), serous cystadenomas, and neuroendocrine tumors of the pancreas (PanNETs) (12–17%). Cystadenomas can occur in the endolymphatic sac, epididymis, and broad ligament [1, 2].

PanNETs are rarely the first manifestation of the syndrome and generally lead to diagnosis suspicion when associated with other typical manifestations of the disease [2]. The majority of PanNETs are incidentally detected lesions by imaging studies performed for other reasons or during the follow up program for VHL syndrome.

**Fig. 1:**
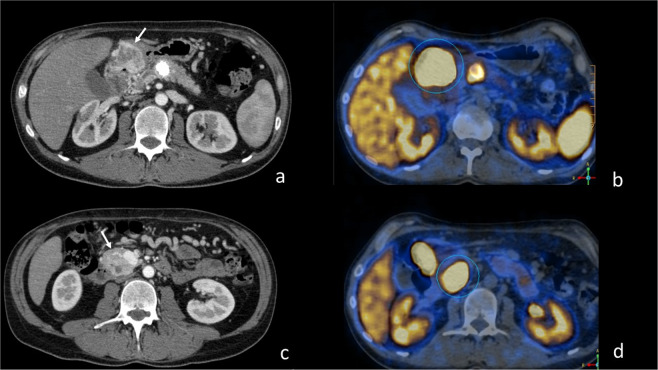
CT scans of multifocal PanNET inhomogeneous hyperdense in arterial phase (**a**, **c**) due to hypervascularization and increased uptake of ^68^Ga-DOTATOC PET/TC (circle in **b** and **d**) involving the stomach (arrow in **a**), the uncinate process of the pancreas (arrow in **c**) and the pancreatic body with an extensively calcified mass

**Fig. 2:**
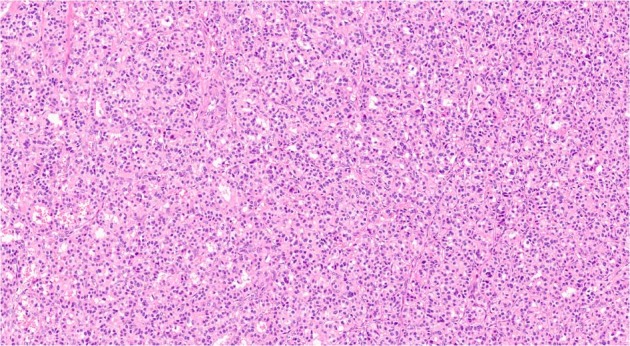
Representative high-magnification field of panNET (Hematoxylin–eosin, ×20 magnification), with solid-trabecular pattern

PanNETs that occur in 12–17% of patients with VHL syndrome are usually nonfunctioning, multiple and with a mean age at presentation two decades earlier compared to their sporadic counterpart [1, 2]. The size of the lesions, generally >3 cm, has been reported as one of main prognostic factors of malignancy. Doubling time <500 days of the lesion suggests an increased metastatic risk too [2–8]. Moreover, a relationship between pancreatic aggressive phenotype and genotype has been described, mainly mutations in exon 3, especially of codons 161/167, suggest an enhanced risk for metastatic PanNETs [6–13]. Conversely, in a French multicenter series, no phenotype correlation with VHL genotype was found in 35 PanNETs, of which 58% was malignant [14]. More recently, in the study by Satoh et al., there was no association between tumor doubling time and exon 3 mutational status and metastatic disease [15].

The revised guidelines for the management of VHL-associated PanNETs recommend surgical resection when PanNETs are >30-mm diameter in the pancreatic body and tail and >20 mm in the pancreatic head and uncinate process [2]. A recent study including a large series of patients collected in the European-American-Asian-VHL-PanNET-Registry, proposed the cutoff diameter of 2.8 cm for every site for surgical indication due to metastatic potential [6].

Given the rarity of the syndrome, literature data regarding the management, the genotype–phenotype correlations and follow-up of PanNETs with VHL syndrome are limited.

The aim of the present study was to analyze clinical presentation, genotype–phenotype correlations, treatment and prognosis of a monocentric series of patients affected by VHL syndrome.

## Patients and methods

We conducted a retrospective analysis of prospectively collected data of VHL patients observed at University of Verona Hospital Trust, ENETS Center of Excellence between January 2005 and January 2020. The study was approved by the Ethics Committee of the University and Hospital Trust of Verona (n. 935 date 16/09/2020).

VHL syndrome was diagnosed in patients with a family history and one typical disorder (CNS/retinal HBA or a main visceral feature, such as RCC, pheocromocytoma, or PanNET) or in those without relevant family history with two or more CNS/retinal HBA or one CNS/retinal HBA and a visceral feature or in cases of positive genetic testing for VHL [1].

The time when the first disease correlated to the syndrome presented was the date chosen as the clinical diagnosis. For all patients, the medical history was collected and previous imaging studies and clinical documentations were examined.

Patients underwent diagnostic imaging procedures to identify the PanNE including: abdomen computed tomography (CT) or magnetic resonance imaging (MRI), ^68^Ga-DOTATOC-PET/CT and ^18^F-fludeoxyglucose (FDG)-PET/CT.

Surgical treatment was performed in all cases with PanNETs > 3 cm in size. The surgical treatment was tailored according to the site of the lesion(s), their number, and the risk of metastatic spread. The procedures adopted were the following: pylorus preserving or Whipple’s procedures (PD), distal spleno-pancreatectomy (DP), or total pancreatectomy (TP). Due to the frequent multiple lesions, the aim of the procedure was to preserve as much pancreatic parenchyma as possible while obtaining oncological radicality.

The diagnosis of PanNET was made through conventional histological and immunohistochemical examinations (chromogranin A, synaptophysin) on surgical specimens. For the non-operated patients, tumor biopsy was based on a transduodenal fine needle aspiration biopsy during endoscopic ultrasound (EUS) or by ^68^Ga-PET-DOTATOC-CT, enhancing somatostatin receptors in the neoplasm. In these cases the CT scan or MRI confirmed the presence of the PanNET on the basis of the peculiar characteristics in pre-contrast and contrast-enhanced images that were different from those of the spleen.

The Ki-67 proliferative index was expressed as a percentage of Ki-67-positive cells in 2000 neoplastic cells in areas of the highest immunostaining using the MIB1 antibody (DBA, Milan, Italy).

Data regarding the site of the NET, histological features according to 2019 WHO classification for PanNETs (G1 < 3%, G2 up to 20%, G3 > 20%), proliferative activity by staining for Ki-67 antigen, presence of local or distant metastases were collected.

All patients attended clinical and imaging study follow-up. Disease-free was based on negative imaging procedures; no significant increase of residual tumor and/or metastases size was defined as stable disease; recurrent disease was defined by new positive imaging procedures after the surgery. The update of follow-up was yearly, or at shorter intervals if deemed necessary.

### Samples and molecular analysis

Genomic DNA from blood was extracted using a column-based purification kit (DNeasy Blood & Tissue kit; Qiagen) and quantified by spectrophotometric and fluorometric analysis using the Qubit DNA HS Assay kit (ThermoFisher), purity and quality was evaluated using NanoDrop ND-2000 [16]. DNA was subjected to Sanger sequencing for samples received before 2016, and to targeted NGS for samples received after 2016.

### Sanger sequencing

Three pairs of oligonucleotide primers were used to amplify all the coding sequence and the exon–intron junctions of the VHL gene (NM_000551). Primers were designed using Internet-based software Primer3-web version 4.0 [17–19] and aligned with the National Center for Biotechnology Information Basic Local Alignment Search Tool using VHL genomic reference sequence (http://www.ncbi.nlm.nih.gov/refseq/rsg; Accession number NC_000003.12 (1014177810153667). The primer sequences were as follows: exon 1: 5′-CCC GGG TGG TCT GGA TCG CG-3′ forward and 5′-GCT ATC GTC CCT GCT GGG TC-3′ reverse; exon 2: 5′-ACC GGT GTG GCT CTT TAA C A-3′ forward and 5′-ACC GGT GTG GCT CTT TAA C A-3′ reverse; exon 3: 5′-GCA AAG CCT CTT GTT CGT TC-3′ forward and 5′-CCA TCA AAA GCT GAG ATG AAA-3′ reverse.

The size of the amplicons was 409 bp for exon 1, 280 bp for exon 2, and 296 bp for exon 3. PCR reactions were performed in a reaction volume of 20 μl containing 0.2-mM dNTP, 2-μl 10X Taq Buffer advance, 0.4-μl 5PRIMETaq DNA Polymerase (Quantabio), 1-μM forward primer, 1-μM reverse primer, and 50-ng DNA. PCR cycling was composed by a single hot start cycle at 95 °C for 3 min, 25 cycles of 94 °C for 20 s, 62 °C for 20 s, and 68 °C for 20 s followed by 3 min of final extension at 68 °C. The PCR products were visualized by the Agilent 2100 Bioanalyzer on-chip electrophoresis (Agilent Technologies) to confirm the presence of the proper molecular weight size products for each exon. Samples were analyzed by direct Sanger sequencing using the ABI Genetic Analyzer 3130XL platform (Applied Biosystems Inc., Foster City, CA), using the Big-Dye terminator version 3.1 Cycle Sequencing kit (Applied Biosystems Inc.) according to the manufacturer specifications. Sequencing results were analyzed by SeqScape software version 1.0 (Applied Biosystems Inc.).

### NGS targeting sequencing

High-coverage sequencing for VHL gene was performed using an AmpliSeq custom panel (TermoFisher) targeting all exons and intron–exon junctions of genes frequently mutated in PanNETs (VHL; RET; MEN1) (Table 1). For each reaction, 20 ng of DNA were used, and the quality of the resulting libraries evaluated by the Agilent 2100 Bioanalyzer on-chip electrophoresis (Agilent Technologies). Sequencing runs were performed on the Ion PGM machine (ThermoFisher) loaded with Ion 318 Chip v3. Base calling, alignment to the hg19 reference genome, and variant calling were done using the Torrent Suite Software v.5.0 (ThermoFisher). Alignments were visually verified with the Integrative Genomics Viewer (IGV). Called variants were annotated using a custom pipeline based on vcflib (https://github.com/ekg/vcflib), the Variant Effect Predictor software [19], and NCBI RefSeq database. Visual verification of alignments on the IGV software v2.3 [20] allowed filtering of variants. This latter step is the key to remove false calls due to technique-dependent mispriming or sample age-related deamination, which cannot be ruled out by automated variant calling and filtering procedures [21].

ClinVar database (https://www.ncbi.nlm.nih.gov/clinvar) was used to assess clinical significance of mutations; which were classified in pathogenic, likely pathogenic, uncertain significance, likely benign, benign according to American College of Medical Genetics and Genomics–Association for Molecular Pathology classification [22].

Although NGS technology differs from the traditional Sanger method in that it analyzes many sequences in parallel, in this context, the two techniques are comparable in terms of accuracy because the three VHL exons and intron–exon junctions were sequenced with both methods.

### Statistical analysis

Results were explicated as means ± SD. It was considered a significant difference when *p* < 0.05. Differences among the groups carrying a statistical significance with *p* < 0.05 were tested with Two-sample Student’s *t* test.

## Results

During the study period, VHL syndrome was diagnosed in 17 patients (7 males and 10 females, 12 probands and 5 relatives) with a mean age at diagnosis of 30.8 ± 18.4 years (range 3–73).

The 13 patients with PanNETs comprised 10 probands (4 males and 6 females) and 3 relatives (1 male and 2 females).

Of the four patients who did not harbor PanNETs, a 3-year-old patient was asymptomatic, three patients had multiple pancreatic cysts, of which two had RCC and one SNC HBA.

The clinical presentation at VHL syndrome diagnosis and the VHL-correlated diseases found during the study period are summarized in Table 1.

**Table 1:** Main characteristics of the 17 patients with VHL syndrome

Characteristics	Number (%)
Mean age at diagnosis, years ± SD	30.8 ± 18.4
Gender, M/F	7/10
VHL diagnosis by	
PanNET	4 (23.5)
Pheocromocytoma	3 (17.6)
HBA CNS/retina	3 (17.6)
Pancreatic cysts	1 (5.8)
RCC	1 (5.8)
VHL clinical/genetic screening	5 (29.4)
VHL manifestations during the study period	
Pancreatic lesions	15 (88.2)
Cysts	2 (11.8)
PanNET	13 (76.5)
HBA	16 (94.1)
CNS	10 (58.8)
Retina	6 (35.3)
Pheocromocytoma	5 (29.4)
RCC	5 (29.4)
Paraganglioma	4 (23.5)

Numbers in parentheses represent percentages unless otherwise noted

PanNETs were found at the time of VHL diagnosis in 4/13 (30.7%) patients and in 9 patients during follow-up after a median time of 12 years (range 1–21). PanNETs were diagnosed at a median age of 37 years (range 24–72). All PanNETs were nonfunctioning and asymptomatic.

All PanNETs were incidentally discovered through imaging procedures performed for nonspecific abdominal pain or during the follow-up. The four patients in whom PanNETs led to VHL diagnosis had presented other manifestations before diagnosis as follows: paraganglioma in one case, pheocromocytoma in one case, CNS HBA in one case, and pancreatic cysts in one case.

Six patients (46.2%) had multiple PanNETs, whereas seven patients (53.8%) had a single tumor. The median size was 25 mm (range 7–60).

Regarding diagnostic procedures, 13 patients performed MRI, 9 patients plus CT, 2 plus EUS with biopsy. These procedures resulted positive in 13 patients affected by PanNETs.

^68^Ga-DOTA-peptide PET/TC was positive in 8/8 and ^18^F-(FDG)-PET/TC in 6/7 (85.7%) of patients who underwent it. Six patients underwent both ^68^Ga-DOTATOC PET/TC and ^18^F-(FDG)-PET/TC of which five had both the procedures positive, whereas one had ^68^Ga-DOTATOTAC PET/TC positive and ^18^F-(FDG)-PET/TC negative. The cutoff of SUV used to define the positivity was ≥4. No tumor with SUV < 4 was defined as FDG-positive tumor (Fig. 1).

### Resected patients

Six out of 13 (46.1%) patients with PanNETs underwent surgery at a median age of 30 years (range 24–40). One patient was operated on after peptide receptor radionuclide therapy that obtained a tumor shrinkage of 15 mm (from 50 to 35 mm).

All patients were treated with open surgery. Whipple’s PD was performed in two cases, pylorus preserving PD in two cases, DP in one case, TP in one case. Regarding complications perioperative mortality was nil, while among postoperative abdominal complications pancreatic fistula occurred in two cases.

### Pathologic features of resected tumors

Pancreatic tumors included a single lesion in three patients, two lesions in two patients, and multiple lesions in one.

The mean diameter of the main lesion was 40 ± 10.9 mm. According to grading, four lesions were G1, two lesions were G2, and none were G3 lesion.

Lymph node metastases were found in four patients (66.7%), none had distant metastases. The median diameter of the PanNET with metastases was 42.5 mm, range 30–60, and two tumors were G1 and two were G2.

The mean tumor diameter of the metastatic PanNETs was significantly larger than the nonmetastatic PanNETs, including those conservatively treated [44.2 ± 9.3 vs. 17.4 ± 7 mm, respectively (p = 0.00049)] (Fig. 2).

### Postoperative follow-up

All patients were alive after a median follow-up of 3 years (range 1–13) from pancreatic surgery.

Five patients were disease-free (83.3%) at the last follow-up. One patient developed multiple liver metastases with the main lesion of 15 mm in the VI segment after 3 years from surgery, which were treated with transcatheter arterial embolization and somatostatin analogs plus sunitinib with stable disease at last follow up.

### Conservatively treated patients

Seven patients with a mean tumor diameter of 15 ± 5.3 mm did not undergo surgery and had stable disease after a median follow-up of 2 years (range 3 months to 11 years).

One patient showed occurrence of an 8-mm PanNET and an increase in size of a pre-existing PanNET (from 10 to 17 mm) at 2-year follow-up.

Clinico-pathological characteristics and surgical procedures of PanNETs with VHL are summarized in Table 2.

**Table 2:** Clinico-pathological characteristics and surgical procedures of PanNETs with VHL

Characteristics	Value
PanNETs *n*	13
Single lesion *n* (%)	7 (53.8)
Multiple lesions *n* (%)	6 (46.2)
Nonfunctioning *n* (%)	13 (100)
Site of main panNET: body-tail *n* (%)	7 (53.8)
Head-uncinate process *n* (%)	6 (46.2)
Lymph node metastases *n* (%)	4 (31)
Distant metastases *n*	0
Resected PanNETs (*n*)	6
PanNET diameter (mm; mean ± SD)	40 (10.9)
Type of pancreatic resection (*n*)	
Whipple’s PD	2
Pylorus preserving PD	2
DP	1
TP	1
G1 (Ki-67 < 3%)	4
G2 (Ki-67 3% ≤ and ≤20%)	2
G3 (Ki-67 > 20%)	0
Median follow-up after surgery (years, range)	3 (1–13)
Non-resected PanNETs (*n*)	7
PanNET diameter (mm; mean ± SD)	15 (5.3)
Median follow-up (years, range)	2 (3 months to 11)

*n*: number of patients

### Genetic analysis and genotype–phenotype correlations

All but one patient underwent germline genetic testing, which resulted positive. One patient, with positive family history and multiple typical manifestations of the disease, refused to perform the genetic testing.

All germline VHL mutations in probands/families affected and related phenotype are summarized in Table 2. Nine different VHL mutations were identified (Table 3). Three (25%) cases had de novo mutations.

**Table 3:** Germline VHL mutations in probands/family member affected patient

Patient	Proband/familiar (P/F)	Exon/intron	Mutation type	Mutation cDNA	Mutation p. protein	Phenotype
1	P	Exon 1	Deletion	c.227_229delTCT	p. Phe76del	HBA CNS, PanNET
2a	P	Exon 1	Missense	c.233A>G	p. Asn78Ser	RCC, HBA CNS/Ret, Pan-Cyst, PanNET
2b	F	“	“	“	“	PPGL, HBA CNS/Ret, RCC, PanNET
4	P	Exon 1	Missense	c.245G>T	p. Arg82Leu	Pheo, HBA CNS, PanNET
5a	P	Exon 1	Missense	c.332G>A	p. Ser111Asn	HBA CNS, Pan/Kid-Cysts
5b	F	“	“	“	“	RCC, Pan/Kid-Cysts
7	P	Intron 1	Splice site	c. 341-3T>G	/	HBA CNS, Metastatic PanNET
8	P	Exon 1	Missense	c.340G>C	p. Gly114Arg	HBA Ret, PanNET
9	P	Exon 3	Nonsense	c.481C>T	p. Arg161Ter	Pan/Kid-Cysts, RCC
10a	P	Exon 3	Missense	c.499C>T	p. Arg167Trp	Pheo, PPGL, HBA CNS/Ret, PanNET, Liver Metastasis
10b	F	“	“	“	“	Pheo, PPGL, HBA Ret, PanNET
10c	F	“	“	“	“	RCC, HBA CNS, RCC, PanNET
10d	F	“	“	“	“	/
14	P	Exon 3	Missense	c.499C>T	p. Arg167Trp	Pheo, HBA CNS, PanNET
15	P	Exon 3	Missense	c.500G>A	p. Arg167Gln	PPGL, Metastatic PanNET
16	P	Exon 3	Missense	c.500G>A	p. Arg167Gln	HBA CNS/Ret, PanNET

*HBA CNS/Ret* central nervous system/retina haemangioblastoma, *PPGL* paraganglioma, *Pheo* pheocromocytoma, *PanNET* neuroendocrine pancreatic tumor, *Pan-Cyst/Kid-Cyst* pancreatic/kidney cyst, *RCC* renal cell carcinoma

/ none, “ same as above

Mutations were missense in 6/9 cases (77.7%), whereas only one nonsense mutation, one deletion, and one splicing mutation were found in the probands.

A genotype–phenotype correlation was not observed, in particular between the presence of the metastases and tumor diameter and the type of mutation.

Among patients with metastatic PanNETs missense, mutations in exon 3 were present in two subjects and a splicing mutation in intron 1, which had been previously reported as of uncertain significance in ClinVar Database, in one case.

All patients with mutations in exon 1 were affected by nonmetastatic PanNETs.

No significant difference in PanNET mean diameter was found between patients with exons 1 and 3 [21.6 ± 7.3 vs. 25.2 ± 14.5 mm, respectively (*p* = 0.57)].

The only patient who developed liver metastases had mutation of 167 codon of exon 3, which has been considered a hotspot for VHL germline mutations associated with enhanced risk for metastatic PanNETs [5].^1^,^2^

## Discussion

The aim of our study was to analyze the clinical presentation, tumor characteristics, treatments and prognosis of a single series of PanNETs associated with VHL syndrome.

The study showed that PanNETs are a frequent manifestation of VHL syndrome, in fact they were discovered in 76.5% of the cases in our series. In more than one third of the patients, PanNET was the disease that led to VHL diagnosis, even though other typical manifestations of the syndrome were already present but not considered to be associated with the syndrome.

This unusual presentation of VHL syndrome and the higher prevalence of PanNETs in our study compared to that reported in the literature, which is up to 17% [10], can be explained by patient recruitment modalities as our hospital is a reference center for PanNETs.

Like those reported in the literature, the PanNETs in our series were nonfunctioning, asymptomatic, often multiple and incidentally detected lesions by imaging studies performed for other reasons or during the follow up of VHL syndrome [1, 13]. However, the prevalence of metastatic PanNETs in our series was 30.7%, which is higher than that reported in the literature of up to 20% [6].

One main open issue is to identify the prognostic factors of aggressiveness of PanNETs in VHL.

The strongest predictor for malignancy is the cutoff diameter >3 cm of the PanNET, which is considered at risk of metastases. In our series a group of patients with small PanNETs with <3-cm diameter were conservatively treated and all but one remained stable after a median follow up of 2 years (range 3 months to 11 years). Patients with tumor diameter >3 cm were operated on. The mean tumor diameter of the metastatic PanNETs was found to be significantly larger than the nonmetastatic PanNETs including those conservatively treated [44.2 ± 9.3 vs. 17.4 ± 7 mm, respectively (*p* = 0.00049)].

Even though the number of patients in our series was limited and follow-up was short, this observation supports the current trend of conservative treatment for only nonfunctioning PanNETs of small diameter (<3 cm), which are reported to be associated with low risk of metastases and mortality. However, given the prevalence of metastases of 31% in the whole series a surgical approach is mandatory in patients with tumor >3 cm and or with increased growth rate.

Regarding tumor grade, as reported in few articles [12–14], in our series, we found only G1 or G2 tumors. Moreover, the sensitivity of Ga-DOTATOC-PET was of 100%, demonstrating the high presence of somatostatin receptors. These observations reflect the relatively indolent behavior of the majority of PanNETs associated with VHL syndrome.

Regarding genotype–phenotype association, even though PanNETs can occur in carriers of mutations of any type, they are more frequent and more aggressive in patients with mutations in exon 3 with hot spots in codons 161/167 [6].

In contrast to Krauss et al. [6] and in line with the study by Corcos et al. [14] and Satoh et al. [15], we did not find a correlation between the somatic mutation and the malignant phenotype of PanNETs.

Two out of the three patients with metastatic PanNET with available genetic testing were carriers of missense mutation in exon 3, at 167 codon, which is reported to be associated with enhanced risk of malignancy. However, another four patients from three different families, carriers of the same germinal mutation, harbored small PanNETs that remained stable during follow up. Therefore, besides the mutation in exon 3, other factors that can contribute to the malignant behavior of the PanNETs such as diameter of the lesion >3 cm and double-timing of the lesion <500 should be considered [11].

In our series, one patient with metastatic PanNET was a carrier of a splicing mutation in intron 1, previously reported as of uncertain significance in ClinVar Database. This germline mutation was also found in the histological specimen of the tumor, demonstrating the pathogenetic role of this mutation of the VHL syndrome.

Four patients out of 17 in our series (23%) carried “de novo” mutations—none of their relatives who underwent genetic testing were affected by the syndrome. This is in line with data from literature, which report a prevalence of around 20% [1, 2, 11].

## Conclusions

PanNETs are common disease of the VHL syndrome and can be the presenting feature. The majority are small, nonfunctioning, and well or moderately differentiated with high expression of somatostatin analog receptors. Tumor diameter rather than genetic mutation is a negative prognostic factor of malignancy.

## Footnotes

1 The significance of " is : same as above.

2 The significance of / is: none.

## References

[1] Lonser RR, Gladys MG, McClellan W, Chew EY, Libutti SK, Marston Linehan W, Oldfield EH (2003). von Hippel–Lindau disease. Lancet.

[2] Charlesworth M, Verbeke CS, Falk GA, Walsh M, Smith AM, Morris-Stiff G (2012). Pancreatic lesions in von Hippel–Lindau disease? A systematic review and meta-synthesis of the literature. J. Gastrointest. Surg..

[3] Crespigio J, Berbel LCL, Dias MA, Berbel RF, Pereira SS, Pignatelli D, Mazzuco TL (2018). Von Hippel–Lindau disease: a single gene, several hereditary tumors. J. Endocrinol. Invest..

[4] Keutgen XM, Hammel P, Choyke PL, Libutti SK, Jonasch E, Kebebew E (2016). Evaluation and management of pancreatic lesions in patients with von Hippel–Lindau disease. Nat. Rev. Clin. Oncol..

[5] Libutti SK, Choyke PL, Bartlett DL, Vargas H, Walther M, Lubensky I, Glenn G, Linehan WM, Alexander HR (1998). Pancreatic neuroendocrine tumors associated with von Hippel-Lindau disease: diagnostic and management recommendations. Surgery.

[6] Krauss T, Ferrara AM, Links TP, Wellner U, Bancos I, Kvachenyuk A, Villar Gómez de Las Heras K, Yukina MY, Petrov R, Bullivant G, von Duecker L, Jadhav S, Ploeckinger U, Welin S, Schalin-Jäntti C, Gimm O, Pfeifer M, Ngeow J, Hasse-Lazar K, Sansó G, Qi X, Ugurlu MU, Diaz RE, Wohllk N, Peczkowska M, Aberle J, Lourenço DM, Pereira MAA, Fragoso MCB, Hoff AO, Almeida MQ, Violante AHD, Quidute ARP, Zhang Z, Recasens M, Díaz LR, Kunavisarut T, Wannachalee T, Sirinvaravong S, Jonasch E, Grozinsky-Glasberg S, Fraenkel M, Beltsevich D, Egorov VI, Bausch D, Schott M, Tiling N, Pennelli G, Zschiedrich S, Därr R, Ruf J, Denecke T, Link KH, Zovato S, von Dobschuetz E, Yaremchuk S, Amthauer H, Makay O, Patocs A, Walz MK, Huber TB, Seufert J, Hellman P, Kim RH, Kuchinskaya E, Schiavi F, Malinoc A, Reisch N, Jarzab B, Barontini M, Januszewicz A, Shah N, Young WF, Opocher G, Eng C, Neumann HPH, Bausch B (2018). Preventive medicine of von Hippel–Lindau disease-associated pancreatic neuroendocrine tumors. Endocr. Relat. Cancer.

[7] Arnon L, Halperin R, Tirosh A (2021). Impact of pancreatic neuroendocrine tumor on mortality in patients with von Hippel–Lindau disease. Endocr. Pract..

[8] Ahmad S, Naber MR, Giles RH, Valk GD, van Leeuwaarde RS (2021). Diagnostic and management strategies for pNETs in Von Hippel–Lindau: a systematic review. Endocr. Relat. Cancer.

[9] M. Falconi, D.K. Bartsch, B. Eriksson, G. Klöppel, J.M. Lopes, J.M. O’Connor, R. Salazar, B.G. Taal, M.P. Vullierme, D. O’Toole, Barcelona Consensus Conference participants, ENETS Consensus Guidelines for the management of patients with digestive neuroendocrine neoplasms of the digestive system: well-differentiated pancreatic non-functioning tumors. Neuroendocrinology **95**, 120–134 (2012) 10.1159/000335587 22261872

[10] Blansfield JA, Choyke L, Morita SY, Choyke PL, Pingpank JF, Alexander HR, Seidel G, Shutack Y, Yuldasheva N, Eugeni M, Bartlett DL, Glenn GM, Middelton L, Linehan WM, Libutti SK (2007). Clinical, genetic and radiographic analysis of 108 patients with Von Hippel–Lindau disease (VHL) manifested by pancreatic neuroendocrine tumors (PANNETs). Surgery.

[11] Nordstrom-O’Brien M, van del Luijt RB, van Rooijen E, van der Ouweland AM, Majoor-Krakauer DF, Lolkema MP, van Brussel A, Voest EE, Giles RH (2010). Genetic analysis of von Hippel–Lindau disease. Hum. Mutat..

[12] Tirosh A, Sadowski SM, Linehan WM, Libutti SK, Patel D, Nilubol N, Kebebew E (2018). Association of VHL genotype with pancreatic neuroendocrine tumor phenotype in patients with von Hippel–Lindau disease. JAMA Oncol..

[13] Gläsker S, Vergauwen E, Koch CA, Kutikov A, Vortmeyer AO (2020). Von Hippel–Lindau disease: current challenges and future prospects. Onco Targets Ther..

[14] Corcos O, Couvelard A, Giraud S, Vullierme MP, O’Toole D, Rebours V, Stievenart JL, Penfornis A, Niccoli-Sire P, Baudin E, Sauvanet A, Levy P, Ruszniewski P, Richard S, Hammel P (2008). Endocrine pancreatic tumors in von Hippel–Lindau disease clinical, histological, and genetic features. Pancreas.

[15] Satoh K, Sadowski SM, Dieckmann W, Quezado M, Nilubol N, Electron K, Dhaval P (2016). 18F-FDG PET/CT volumetric parameters are associated with tumor grade and metastasis in pancreatic neuroendocrine tumors in von Hippel–Lindau disease. Ann. Surg. Oncol..

[16] Simbolo M, Gottardi M, Corbo V, Fassan M, Mafficini A, Malpeli G, Lawlor RT, Scarpa A (2013). DNA qualification workflow for next generation sequencing of histopathological samples. PLoS ONE.

[17] Untergasser A, Cutcutache I, Koressaar T, Ye J, Faircloth BC, Remm M, Rozen SG (2007). Primer3-new capabilities and interfaces. Nucleic Acids Res..

[18] Koressaar T, Remm M (2007). Enhancements and modifications of primer design program Primer3. Bioinformatics.

[19] McLaren W, Pritchard B, Rios D, Chen Y, Flicek P, Cunningham F (2010). Deriving the consequences of genomic variants with the Ensembl API and SNP effect predictor. Bioinformatics.

[20] Thorvaldsdottir H, Robinson JT, Mesirov JP (2013). Integrative genomics viewer (IGV): high-performance genomics data visualization and exploration. Brief. Bioinform..

[21] Simbolo M, Vicentini C, Mafficini A, Fassan M, Pedron S, Corbo V, Mastracci L, Rusev B, Pedrazzani C, Landoni L, Grillo F, Cingarlini S, Rindi G, Luchini C, Scarpa A, Lawlor RT (2018). Mutational and copy number asset of primary sporadic neuroendocrine tumors of the small intestine. Virchows Arch..

[22] Nykamp K, Anderson M, Powers M, Garcia J, Herrera B, Ho YY, Kobayashi Y, Patil N, Thusberg J, Westbrook M, Topper S, Invitae Clinical Genomics Group (2017). Sherloc: a comprehensive refiement of the ACMGMP variant classification criteria. Genet. Med..

